# Spike protein mutations and structural insights of pangolin lineage B.1.1.25 with implications for viral pathogenicity and ACE2 binding affinity

**DOI:** 10.1038/s41598-023-40005-y

**Published:** 2023-08-12

**Authors:** Shahina Akter, Jonas Ivan Nobre Oliveira, Carl Barton, Murshed Hasan Sarkar, Muhammad Shahab, Tanjina Akhtar Banu, Barna Goswami, Eshrar Osman, Mohammad Samir Uzzaman, Tasnim Nafisa, Maruf Ahmed Molla, Mahmuda Yeasmin, Maisha Farzana, Ahashan Habib, Aftab Ali Shaikh, Salim Khan

**Affiliations:** 1 Bangladesh Council of Scientific and Industrial Research (BCSIR), Dhaka, Bangladesh; 2 SciTech Consulting and Solutions, Dhaka, Bangladesh; 3 National Institute of Laboratory Medicine and Referral Center, Dhaka, Bangladesh; 4 Birkbeck, University of London, Malet St, Bloomsbury, London, WC1E 7HX UK; 5 Department of Chemistry, University of New Brunswick, Fredericton, NB E3B 5A3 Canada; 6 State Key Laboratories of Chemical Resources Engineering, Beijing University of Chemical Technology, Beijing, 100029 China; 7 Department of Biophysics and Pharmacology, Bioscience Center, Federal University of Rio Grande do Norte, Natal, RN 59078-900 Brazil; 8 SUNY Upstate Medical University, Syracuse, NY 13207 USA; 9 King’s College London, London, England

**Keywords:** Biochemistry, Computational biology and bioinformatics, Microbiology

## Abstract

Severe Acute Respiratory Syndrome Coronavirus 2 (SARS-CoV-2), the causative agent of COVID -19, is constantly evolving, requiring continuous genomic surveillance. In this study, we used whole-genome sequencing to investigate the genetic epidemiology of SARS-CoV-2 in Bangladesh, with particular emphasis on identifying dominant variants and associated mutations. We used high-throughput next-generation sequencing (NGS) to obtain DNA sequences from COVID-19 patient samples and compared these sequences to the Wuhan SARS-CoV-2 reference genome using the Global Initiative for Sharing All Influenza Data (GISAID). Our phylogenetic and mutational analyzes revealed that the majority (88%) of the samples belonged to the pangolin lineage B.1.1.25, whereas the remaining 11% were assigned to the parental lineage B.1.1. Two main mutations, D614G and P681R, were identified in the spike protein sequences of the samples. The D614G mutation, which is the most common, decreases S1 domain flexibility, whereas the P681R mutation may increase the severity of viral infections by increasing the binding affinity between the spike protein and the ACE2 receptor. We employed molecular modeling techniques, including protein modeling, molecular docking, and quantum mechanics/molecular mechanics (QM/MM) geometry optimization, to build and validate three-dimensional models of the S_D614G-ACE2 and S_P681R-ACE2 complexes from the predominant strains. The description of the binding mode and intermolecular contacts of the referenced systems suggests that the P681R mutation may be associated with increased viral pathogenicity in Bangladeshi patients due to enhanced electrostatic interactions between the mutant spike protein and the human ACE2 receptor, underscoring the importance of continuous genomic surveillance in the fight against COVID -19. Finally, the binding profile of the S_D614G-ACE2 and S_P681R-ACE2 complexes offer valuable insights to deeply understand the binding site characteristics that could help to develop antiviral therapeutics that inhibit protein–protein interactions between SARS-CoV-2 spike protein and human ACE2 receptor.

## Introduction

Severe acute respiratory syndrome coronavirus 2 (SARS-CoV-2) is the virus responsible for the global pandemic known as coronavirus disease 2019 (COVID-19), which emerged in December 2019. The World Health Organization (WHO) reports that COVID-19 has caused significant morbidity and mortality worldwide, with 651 million reported cases and 6.6 million deaths as of December 2022 (WHO Coronavirus (COVID-19) Dashboard|WHO Coronavirus (COVID-19) Dashboard with Vaccination Data).

In Bangladesh, the first case of COVID-19 was reported in March 2020, and since then, the country has experienced a steady increase in infections^1^. As of December 30, 2022, there have been 2,037,024 confirmed cases and 29,439 deaths (https://corona.gov.bd/?gclid). Throughout the pandemic, samples from COVID-19 positive patients were collected from the National Institute of Laboratory Medicine and Referral Center (NILMRC) and sent to the Bangladesh Council of Scientific and Industrial Research (BCSIR) for sequencing SARS-CoV-2 isolates. The generated data were subsequently submitted to the Global Initiative for Sharing All Influenza Data (GISAID) and the National Center for Biotechnology Information/EUA (NCBI). Whole-genome sequencing was performed on these isolates to analyze the mutations and understand their relationship to the original SARS-CoV-2 strain in Wuhan, China^2,3^.

The spike (S) protein of SARS-CoV-2 plays a crucial role in viral entry into host cells. This glycoprotein forms trimers on the virion surface and binds to the host-cell receptor angiotensin-converting enzyme 2 (ACE2) through its receptor-binding domain (RBD). Subsequent structural rearrangements facilitate the fusion of the viral membrane with the host-cell membrane^4^. The S protein has undergone significant evolutionary changes, leading to the emergence of various variants of concern. These variants exhibit multiple mutations in the spike protein and can impact viral replication efficiency, neutralizing antibody sensitivity, and binding affinity to the ACE2 receptor^5^.

The Delta variant, in particular, has demonstrated higher replication efficiency and reduced sensitivity to neutralizing antibodies compared to the original strain^6^. It exhibits increased binding affinity to the ACE2 receptor and enhanced spike-mediated entry efficiency, making it highly concerning^7,8^.

In this study, we aim to identify the prevalent variants and characteristic mutations of SARS-CoV-2 in Bangladesh throughout 2021. We will investigate the impact of these mutations on the spike protein by analyzing the interaction patterns between mutant spike proteins and the ACE2 receptor. Additionally, we will assess changes in protein dynamics and stability resulting from vibrational entropy alterations. To achieve these objectives, we will employ various molecular modeling techniques, including protein modeling, molecular docking, and geometry optimization using quantum mechanics/molecular mechanics (QM/MM), and protein stability analysis.

By comprehensively analyzing the genetic variations and their effects on the spike protein, we hope to contribute to the understanding of SARS-CoV-2 evolution in Bangladesh and its implications for the ongoing COVID-19 pandemic.

## Methods

### Whole-genome sequencing

SARS-CoV-2 detection via RT-PCR assay (Novel Coronavirus (2019-nCoV) Nucleic Acid Diagnostic Kit, Sansure Biotech) from nasopharyngeal specimens of 17 COVID-19-positive patients collected from the NILMRC, Bangladesh. Library preparation and next generation sequencing of the samples were conducted by the Genomic Research Laboratory of BCSIR. This study was approved by the human research ethics committee of NILMRC (project code: 224125200). All methods were performed in accordance with the relevant guidelines and regulations. Informed consent was obtained from all subjects and/or their legal guardian(s).

Viral RNA was extracted using PureLink™ Viral RNA/DNA Mini Kit (Cat. no: 12280050, Thermofisher Scientific, USA)^1^. The cDNA of all samples was used to prepare paired-end libraries with the Nextera ™ DNA Flex Library Preparation kit according to the manufacturer's instructions (Illumina Inc., San Diego, CA). The Library pool of the samples was sequenced using the S4 flow cell of Illumina NextSeq 550 instrument in a paired-end fashion (read length 151 bp; Illumina Inc.). FASTQ files were generated in the Illumina BaseSpace Sequence Hub (https://basespace.illumina.com). The generated data for BCSIR-NILMRC-422 and BCSIR-NILMRC-424 after NGS sequencing were submitted to the NCBI as SRA submissions and got the SRA numbers SRP336906 and SRP336782 (PRJNA762998 and PRJNA762670) respectively. Genomes of SARS-CoV-2 viruses were assembled using Basespace DRAGEN RNA Pathogen Detection V3.5.14 software with default parameters. Consensus FASTA files of the SARS-CoV-2 genome were generated using Basespace Dragon RNA Pathogen Detection software version 3.5.1 (https://basespace.illumina.com) with default settings. The sequences of SARS-CoV-2 isolates from Bangladeshi patients were compared to the reference SARS-CoV-2 sequence (NC_045512.1), through nucleotide substitutions. The positions of the nucleotides and amino acids were further confirmed from GeneBank reference sequences (NC_045512.1)^9^.

### Design, refinement, and validation of the tertiary structure of the mutated spike proteins

Genome assembly of the raw data was performed using the assembly toolkit in the EzCOVID19 cloud service, provided on the EzBioCloud website (https://eztaxon-e.ezbiocloud.net). Genome sequences within the assembly were all 29,903 nucleotides in length and covered 99.84% of the SARS-CoV-2 Wuhan reference genome (NC_045512.2), with an average GC content of 38%. The FASTA file containing the sequences of all spike proteins was aligned and visualized using CLUSTAL Omega (https://www.ebi.ac.uk/Tools/msa/clustalo/) and Mview (https://www.ebi.ac.uk/Tools/msa/mview/).

CFSSP (http://www.biogem.org/tool/chou-fasman/), Swiss-Model (https://swissmodel.expasy.org/), and RaptorX (http://raptorx.uchicago.edu/) servers were used to predict secondary and 3D structure as well as solvent accessibility and disordered regions of spike proteins with D614G (S_D614G) and P681R (S_P681R) mutations. The latter one uses an input sequence in FASTA format and predicts its tertiary structure, based on three strategies: (a) single and (b) multiple template threading and (c) alignment quality prediction. To verify the quality of the predicted 3D structure, the server assigns confidence scores, including the P-value for relative global quality, GDT (Global Distance Test) and uGDT (un-normalized GDT) for absolute global quality. The RaptorX-Contact was officially ranked 1st in contact prediction in terms of F1 score in the worldwide protein structure prediction (CASP) competition round XII.

The 3D models obtained for S-D614G and S-P681R were refined using the GalaxyRefine server (http://galaxy.seoklab.org/). This web server is based on a method that refines 3D structures by implementing short Molecular Dynamics (MD) after repeated side-chain repacking perturbations at global and local levels, enabling larger movement. This server offers one of the best solutions for improving both local and global quality of the given structure by using the most up to date protein structure predictions available^10^.

The Swiss Model’s Structure Assessment (https://swissmodel.expasy.org/assess), MolProbity (http://molprobity.biochem.duke.edu/index.php), ProSA-web (https://prosa.services.came.sbg.ac.at/prosa.php), SAVES-ERRAT, Verify3D and PROCHECK (https://saves.mbi.ucla.edu/) tools checked the quality and possible errors of the 3D models using a newly validated structural validation protocol^11,12^. Swiss Model’s Structure Assessment Tool was used to obtain information at global and local levels of structure through its own methods (QMEAN and Ramachandran plot) or by running additional software tools (MolProbity). The ProSA-web tool calculates an overall quality score for each input protein structure and highlights any problematic regions using a 3D molecule viewer. If the calculated score falls outside the range characteristic of native proteins, the structure likely contains errors^13^. The SAVES server v6.0 generated a Ramachandran plot to visualize the energetically allowed and disallowed dihedral angles psi (ψ) and phi (φ) of the amino acids^14^, as well as the overall quality score of the modeled protein by analyzing the unbound atom–atom interactions, and compared it with reliable high-resolution crystal structures using ERRAT, Verify3D, Prove, PROCHECK, and WHATCHECK.

### Protein motion and flexibility analysis

Proteins are dynamic macromolecules whose function is closely linked to their biological movements^15^. It is known that both drug-resistant and genetic disease mutations can act through changes in the conformational balance and dynamics of proteins^16^. To predict the effects of single-point mutations on S protein stability, we used the DynaMut server (http://biosig.unimelb.edu.au/dynamut/) to analyze the protein movement and flexibility of S_D614G and S_P681R. In this tool, ΔΔG ≥ 0 is considered stabilizing, and ΔΔG < 0 is considered destabilizing. In the entire blinded set of 702 mutations, including both forward and hypothetical reverse mutations, DynaMut achieved performance compatible with (or better than) that of established methods^17^.

### Molecular docking and QM:MM simulations

Using the individual structures of two molecules of interest, a molecular docking tool identifies the binding modes through a search algorithm and evaluates them with an energy scoring function, thus being able to predict protein–protein interactions. Here, initially, the molecular structures of protein_1/ligand and protein_2/receptor ((S_D614G and S_P681R) ACE2-PDB ID: 7W9I) were adjusted by adding charges (protonation or deprotonation) in atoms and correcting bonds, using the PROPKA 3.1 package (https://github.com/jensengroup/propka/). The pH parameter used was within the physiological range (7.2–7.4) due to the presence of MAYV in the bloodstream. To make the calculation more accurate, a force field called CHARMm (Chemistry at Harvard Molecular Mechanics) version 36, specifically designed for organic molecules, was used to perform molecular dynamics simulations and optimize the geometry of hydrogen atoms^18–21^.

The docking between human ACE2 and spike proteins S_D614G and S_P681R was implemented using Cluspro (https://cluspro.bu.edu). The server performs three computational steps as follows: (1) rigid-body docking by sampling billions of conformations; (2) root-mean-square deviation (RMSD)-based clustering of the 1000 lowest-energy structures generated, to find the largest clusters that will represent the most likely models of the complex; and (3) refinement of selected structures using energy minimization^22^. The best Cluspro model was subjected to an additional refinement step in the HADDOCK interface (https://bianca.science.uu.nl/haddock2.4/refinement/1). This server provides a list of clusters in order of score and presents detailed statistics showing the average score of the top four structures for each cluster^23^.

We used the combined quantum mechanics/molecular mechanics technique (QM/MM) to optimize the geometry of the docking models and to select the most relevant protein–protein complexes from the docking calculations, namely S_D614G-ACE2 and S_P681R-ACE2. The QM /MM methods have now established themselves as the most advanced computational methods for biomolecular systems, justifying the rapidly growing number of publications^24,25^. The approach combines the reaction center/active site as the QM region/layer (ACE2), whereas the MM region/layer accommodates the remaining part of the system (major amino acid residues of the S protein). As most biomolecular events concern the reaction center and not the full biomolecular system, QM/MM is ideal for obtaining relevant insights^26,27^. Here, the popular B3LYP hybrid functional (Becke, three parameters, Lee–Yang–Parr) of exchange–correlation and basis set 6-311G (d, p) were used to expand the electronic orbitals for the QM layer, and all amino acid residues within a radius of 6.0 Å from the centroid of the S protein were allowed to move during geometry optimization.

The best Cluspro and QM/MM models were run using the PRODIGY tool (https://bianca.science.uu.nl//prodigy/lig) for a comparative analysis of binding energies (van der Walls, electrostatic, and desolvation energies). PRODIGY predicts protein–protein binding affinity (or binding free energy) based on the functional and structural features of the biological system, i.e., the interfacial contact network^28^. The RMSD calculation of these same structures compared to their respective original PatchDock complexes was performed by Discovery Studio (https://discover.3ds.com/discovery-studio-visualizer-download). Finally, binding poses and the presence of intermolecular interactions were evaluated using LigPlot+ (https://www.ebi.ac.uk/thornton-srv/software/LigPlus/), PoseView (https://proteins.plus/), and Discovery Studio Visualizer, specifically intermolecular hydrogen bonds (carbon, conventional, and pi-donor H-bonds), electrostatics (salt bridge, attractive charges, pi-cation, pi-anion).

### Ethics approval

This study includes experiments with humans and the human research ethics committee of the National Institute of Laboratory Medicine and Referral Center (NILMRC) approved the whole-genome sequencing of SARS-CoV-2 in this study (project code- 224125200).

### Consent for publication

The submitting research article “Spike Protein Mutations and Structural Insights of Pangolin Lineage B.1.1.25: Implications for Viral Pathogenicity and ACE2 Binding Affinity” for publication in your journal of repute, is a unique article and nobody did it earlier. Consents were taken from all the participates for this publication.

### Preprint

A preprint of the manuscript has previously been published in Research Square, 10.21203/rs.3.rs-1480075/v1.

## Results

A total of 850 SARS-CoV-2 strains collected from different divisions of Bangladesh were subjected to whole-genome sequencing using NGS (NextSeq 550) at BCSIR. The generated sequencing data were submitted to GISAID. In this study, we focused on the analysis of seventeen specific cases, namely hCoV-19/Bangladesh/BCSIR-NILMRC_392, hCoV-19/Bangladesh/BCSIR-NILMRC_398, hCoV-19/Bangladesh/BCSIR-NILMRC_376A, hCoV-19/Bangladesh/BCSIR-NILMRC_390, hCoV-19/Bangladesh/BCSIR-NILMRC_391, hCoV-19/Bangladesh/BCSIR-NILMRC_393, hCoV-19/Bangladesh/BCSIR-NILMRC_396, hCoV-19/Bangladesh/BCSIR-NILMRC_397, hCoV-19/Bangladesh/BCSIR-NILMRC_411, hCoV-19/Bangladesh/BCSIR-NILMRC_420, hCoV-19/Bangladesh/BCSIR-NILMRC_421, hCoV-19/Bangladesh/BCSIR-NILMRC_422, hCoV-19/Bangladesh/BCSIR-NILMRC_423, hCoV-19/Bangladesh/BCSIR-NILMRC_424, hCoV-19/Bangladesh/BCSIR-NILMRC_426, hCoV-19/Bangladesh/BCSIR-NILMRC_427, and hCoV-19/Bangladesh/BCSIR-NILMRC_428.

For all seventeen cases, nearly complete genomes consisting of 29,903 nucleotides were successfully obtained. Based on the analyses conducted using GISAID, these 17 isolates were categorized under the GR clade. Furthermore, Nextclade analysis classified them under the 2B clade. Among the analyzed isolates, hCoV-19/Bangladesh/BCSIR-NILMRC_376A and hCoV-19/Bangladesh/BCSIR-NILMRC_390 were found to belong to lineages other than B.1.1.25 (Table 1). However, the remaining isolates were identified as part of the B.1.1.25 lineage, which is considered a lineage specific to Bangladesh. The parent lineage of B.1.1.25 is B.1.1, as documented in the lineage classification (https://cov-lineages.org/lineage.html?lineage=B.1.1.25)^29^.

**Table 1:** Technical metadata of 17 SARS-CoV2 isolates from Bangladeshi patients.

Virus name	Accession ID	Sequencing technology	Assembly method	Lineage (GISAID)	Clade
hCoV-19/Bangladesh/BCSIR-NILMRC_392	EPI_ISL_603221	NextSeq 550	DRAGEN RNA Pathogen Detection 3.5.15	B.1.1.25	GR
hCoV-19/Bangladesh/BCSIR-NILMRC_398	EPI_ISL_603222	NextSeq 550	DRAGEN RNA Pathogen Detection 3.5.15	B.1.1.25	GR
hCoV-19/Bangladesh/BCSIR-NILMRC_376A	EPI_ISL_603223	NextSeq 550	DRAGEN RNA Pathogen Detection 3.5.15	B.1.1	GR
hCoV-19/Bangladesh/BCSIR-NILMRC_390	EPI_ISL_603224	NextSeq 550	DRAGEN RNA Pathogen Detection 3.5.15	B.1.1	GR
hCoV-19/Bangladesh/BCSIR-NILMRC_391	EPI_ISL_603225	NextSeq 550	DRAGEN RNA Pathogen Detection 3.5.15	B.1.1.25	GR
hCoV-19/Bangladesh/BCSIR-NILMRC_393	EPI_ISL_603238	NextSeq 550	DRAGEN RNA Pathogen Detection 3.5.15	B.1.1.25	GR
hCoV-19/Bangladesh/BCSIR-NILMRC_396	EPI_ISL_603239	NextSeq 550	DRAGEN RNA Pathogen Detection 3.5.15	B.1.1.25	GR
hCoV-19/Bangladesh/BCSIR-NILMRC_397	EPI_ISL_603240	NextSeq 550	DRAGEN RNA Pathogen Detection 3.5.15	B.1.1.25	GR
hCoV-19/Bangladesh/BCSIR-NILMRC_411	EPI_ISL_603241	NextSeq 550	DRAGEN RNA Pathogen Detection 3.5.15	B.1.1.25	GR
hCoV-19/Bangladesh/BCSIR-NILMRC_420	EPI_ISL_603242	NextSeq 550	DRAGEN RNA Pathogen Detection 3.5.15	B.1.1.25	GR
hCoV-19/Bangladesh/BCSIR-NILMRC_421	EPI_ISL_603243	NextSeq 550	DRAGEN RNA Pathogen Detection 3.5.15	B.1.1.25	GR
hCoV-19/Bangladesh/BCSIR-NILMRC_422	EPI_ISL_603244	NextSeq 550	DRAGEN RNA Pathogen Detection 3.5.15	B.1.1.25	GR
hCoV-19/Bangladesh/BCSIR-NILMRC_423	EPI_ISL_603245	NextSeq 550	DRAGEN RNA Pathogen Detection 3.5.15	B.1.1.25	GR
hCoV-19/Bangladesh/BCSIR-NILMRC_424	EPI_ISL_603246	NextSeq 550	DRAGEN RNA Pathogen Detection 3.5.15	B.1.1.25	GR
hCoV-19/Bangladesh/BCSIR-NILMRC_426	EPI_ISL_603247	NextSeq 550	DRAGEN RNA Pathogen Detection 3.5.15	B.1.1.25	GR
hCoV-19/Bangladesh/BCSIR-NILMRC_427	EPI_ISL_603249	NextSeq 550	DRAGEN RNA Pathogen Detection 3.5.15	B.1.1.25	GR
hCoV-19/Bangladesh/BCSIR-NILMRC_428	EPI_ISL_603250	NextSeq 550	DRAGEN RNA Pathogen Detection 3.5.15	B.1.1.25	GR

The age range of the patients was of 22–74 years (Supplementary Fig. 1a), and the sex distribution was 88.23% male and 11.76% female (Supplementary Fig. 1b). To identify clusters within the diversity of SARS-CoV-2, the coronavirus typing tool applies phylogenetic analysis (Supplementary Fig. 2). This typing tool has prudently selected reference sequences that denote the diversity of each well-defined phylogenetic cluster (detailed in nextstrain). Additionally, this tool executed extensive testing to be sure that their reference strains accurately classified other sequences^30,31^. If the sequence is identified as being part of the SARS-CoV-2 cluster, an additional phylogenetic analysis is performed to identify one of the following lineages: *B.1.351_501Y.V2_20H (Beta variant)*: A variant of concerns first originated in South Africa (report-COVID-19 B.1.351 (501Y.V2) Variant of Concern—What We Know So Far; Public Health Ontario, 02.07.2021); *B.1.1.7_501.V1_201*: A cluster of concerns first discovered in the UK by A. Rambaut *et al*. (report) *P.1 aka 501Y.V3 aka 20J*: A cluster of concerns first discovered in Brazil by N. Faria *et al*. (report); *Y453F.*Cluster5_20B: A cluster of concern first discovered in Denmark, believed to have spread from mink infections;* B.1.1.70_501Y_20B *^32^*.*

Assembly was performed by aligning reads to a pre-defined Wuhan reference genome (NC_045512.2). Genome assembly of the raw data was done by the Assembly Toolkit in the EzCOVID19 and EDGE COVID-19 cloud services provided on the EzBioCloud website and the EDGE bioinformatics website, respectively^33,34^. To retain the exceptional variations of raw data using the EDGE COVID-19 cloud service, a consensus genome was generated^33^. After that, the consensus genome was compared to the same reference genome in order to calculate single nucleotide variations (SNVs) with positions. The SNVs were compared against GISAID clade variation markers. The genome presented here belongs to the B.1.1.25 lineage of the GR clade. This result was again confirmed by analyzing the raw data using the default parameters of the coronavirus typing tool offered by Genome Detective^35^.

Table 2 shows the single nucleotide variations, non-synonymous and synonymous mutations, between the input assembly and the SARS-CoV-2 reference NC_045512.2 (https://www.ncbi.nlm.nih.gov/nuccore/NC_045512.two) according to analyzes in the EZBioCloud database (https://www.ezbiocloud.net/tools/sc2/?id=4024a5b2-40bb-40b5-acd6-c4dd65b38d76). The queried dataset covers 99.84% of the SARS-CoV-2 genomic sequence. The nucleotide sequences were indicated starting from the 5′ UTR, while the corresponding amino acid changes were mentioned separately for each protein-coding region. In more than 30% of the viral isolates, nucleotide substitutions of C > T were observed, whereas, G > T and G > A showed 20% and 15% respectively. Among these mostly seen substitutions, C > T, which are present in the ORF1ab, S gene, and ORF3a gene (Table 2). G > T is found in ORF1ab, ORF3a, and the M gene, whereas, G > A present in the S gene and maximum cases in the N gene.

**Table 2:** Common nucleotide substitutions in 17 SARS-CoV-2 viral genomes from Bangladesh (submitted to GISAID in October 2020) compared to the SARS-CoV-2 NCBI reference genome NC 045,512.1.

Nucleotide substitution at the given position	Coverage	Gene	SNV type	Codon		Amino acid		Variant marker
				Assem	Ref.	Assem	Ref.	
C241T	1		5'UTR SNP	TGT	CGT	C	R	G/GH/GR/GV
A1163T	1	ORF1ab	Nonsynonymous	TTT	ATT	F	I	
C2363T	1	ORF1ab	Nonsynonymous	TTT	CTT	F	L	
C3037T	1	ORF1ab	Synonymous	TTT	TTC	F	F	G/GH/GR/GV
T4174A	1	ORF1ab	Synonymous	ACA	ACT	T	T	
G11083T	1	ORF1ab	Nonsynonymous	TTT	TTG	F	L	V
C14408T	1	ORF1ab	Nonsynonymous	CTT	CCT	L	P	
G20238T	1	ORF1ab	Nonsynonymous	AGT	AGG	S	R	
C22120T	1	S	Synonymous	TTT	TTC	F	F	
A23403G	1	S	Nonsynonymous	GGT	GAT	G	D	G/GH/GR/GV
T23599A	1	S	Nonsynonymous	AAA	AAT	K	N	
C25904T	1	ORF3a	Nonsynonymous	TTA	TCA	L	S	
G26211T	1	ORF3a	Synonymous	GTT	GTG	V	V	
G26828T	1	M	Synonymous	CTT	CTG	L	L	
G28580A	1	N	Nonsynonymous	AAT	GAT	N	D	
G28881A	1	N	Nonsynonymous	AAA	AGG	K	R	
G28882A	1	N	Nonsynonymous	AAA	AGG	K	R	GR
G28883C	1	N	Nonsynonymous	CGA	GGA	R	G	
C29578A	1	ORF10	Nonsynonymous	TTA	TTC	L	F	

The viral genes were identified according to the reference sequence information from GeneBank. The nucleotide sequences were indicated starting from the 5’ UTR, while the corresponding amino acid changes were mentioned separately. (https://www.ezbiocloud.net/tools/sc2/?id=4024a5b2-40bb-40b5-acd6-c4dd65b38d76).

The emergence of mutations in SARS-CoV-2 evolution, in the context of ‘variants of concern’, has been characterized by factors that influence virus characteristics, including transmissibility and antigenicity. These mutations are most likely a result of the human population's changing immune profile^36^. The amino acid change D614G in spike protein was noted to be increasing in frequency in April 2020 and to have emerged several times in the global SARS-CoV-2 population^36^. The high dN/dS ratio indicates positive selection at codon position 614, which could confer a moderate advantage for infectivity and transmissibility^35,37^.

Most of the viral isolates were found to have different amino acid substitution combinations (Table 3). However, the same amino acid substitution combinations (Spike_D614G and Spike_P681R) were observed for the samples BCSIR-NILMRC_393, BCSIR-NILMRC_396, BCSIR-NILMRC_421, BCSIR-NILMRC_422, and BCSIR-NILMRC_424. In this study, we have emphasized these two amino acid mutations, and hence we will discuss these two amino acid mutations in detail later on. In every isolate, Spike_D614G is the common mutation found in the spike protein (Table 3). The analyzed viral isolates were found to harbor 11 to 19 mutations per isolate, compared to the reference sequence.

**Table 3:** Amino acid substitutions in all 17 isolates of SARS-CoV2 viruses isolated in Bangladesh.

Virus name	Accession ID	No. of nucleotide mutations	AA substitutions
BCSIR-NILMRC_392	EPI_ISL_603221	19	Spike_N679K, Spike_D614G
BCSIR-NILMRC_398	EPI_ISL_603222	19	Spike_K854N, Spike_T29I, Spike_D614G
BCSIR-NILMRC_376	EPI_ISL_603223	11	Spike_D614G
BCSIR-NILMRC_390	EPI_ISL_603224	14	Spike_D614G
BCSIR-NILMRC_391	EPI_ISL_603225	14	Spike_D614G
BCSIR-NILMRC_393	EPI_ISL_603238	13	Spike_P681R, Spike_D614G
BCSIR-NILMRC_396	EPI_ISL_603239	15	Spike_P681R, Spike_D614G
BCSIR-NILMRC_397	EPI_ISL_603240	12	Spike_D614G
BCSIR-NILMRC_411	EPI_ISL_603241	15	Spike_D614G
BCSIR-NILMRC_420	EPI_ISL_603242	13	Spike_D614G
BCSIR-NILMRC_421	EPI_ISL_603243	13	Spike_P681R, Spike_D614G
BCSIR-NILMRC_422	EPI_ISL_603244	12	Spike_P681R, Spike_D614G
BCSIR-NILMRC_423	EPI_ISL_603245	13	Spike_D614G
BCSIR-NILMRC_424	EPI_ISL_603246	21	Spike_P681R, Spike_D614G
BCSIR-NILMRC_426	EPI_ISL_603247	17	Spike_D614G
BCSIR-NILMRC_427	EPI_ISL_603249	14	Spike_I68T, Spike_D614G, Spike_D1118Y
BCSIR-NILMRC_428	EPI_ISL_603250	13	Spike_D614G, Spike_M1229I

For B.1.1.25 lineage reports with S:P681R study, we have used the outbreak.info tool (https://outbreak.info/situation-reports). The prevalence of the B.1.1.25 lineage with S:P681R in the world, how it is changing over time, and how its prevalence varies across different locations, especially in Bangladesh, have been outlined in this report. To describe the prevalence of sets of mutations in Mutation Situation Reports, we rely on shared virus sequences from the GISAID. We also rely on the accuracy of the sequences and sample metadata deposited in GISAID, while we apply filters to eliminate some low-quality sequences and unreasonable metadata. The mentioned sequences are a sample of the entire number of samples, a biased sample that may not represent the true prevalence of the mutations in the population very frequently. Overall, it is important to note that case numbers for any given lineage/mutation can be significantly affected by total case numbers and rates of genomic sequencing at any particular location (https://outbreak.info/situation-reports)^38^.

**Figure 1:**
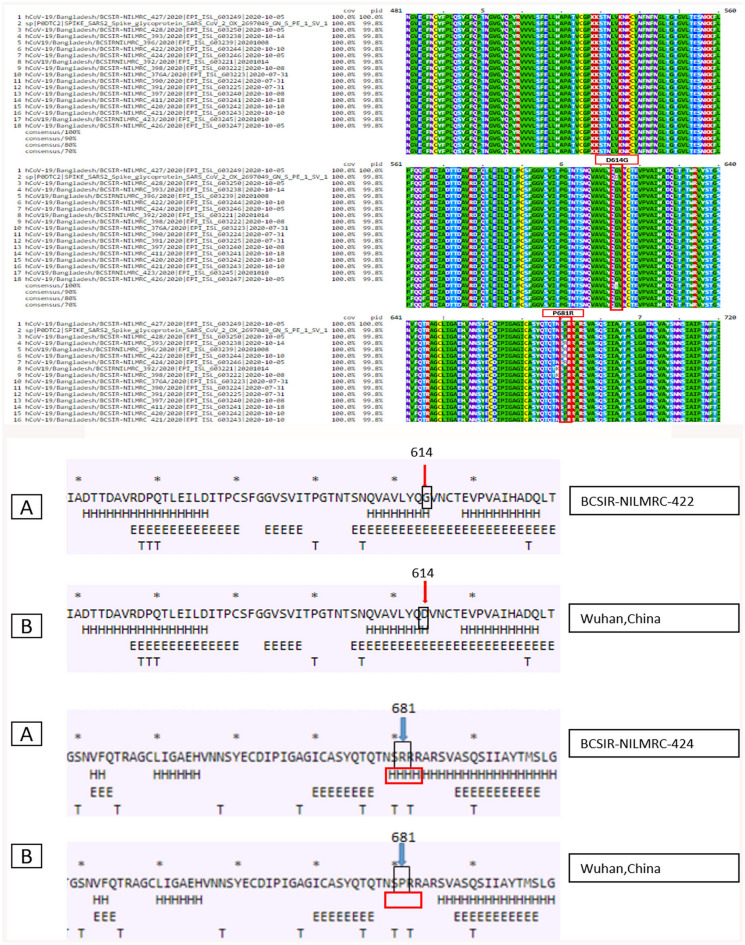
(i) Mutation analysis of 17 isolates from Bangladeshi patients. MSA of Spike protein sequence of Bangladeshi isolates with sequences obtained from Wuhan. Sites of mutation are shown in Red. (ii) Effect of mutation at positions 614 and 681 on the secondary structure of Spike protein in an isolate from Bangladeshi patient, highlighting the area around the residue 614 and 681.

Lineage B.1.1.25 is a current Bangladeshi lineage, and B.1.1 is its parent lineage. Bangladesh (50.0%), Canada (27.0%), the United States of America (11.0%), the United Kingdom (5.0%), and Australia (2.0%) are the most common countries of this lineage. All the spike protein sequences, along with the reference sequence from Wuhan, were aligned using the multiple sequence alignment (MSA) platform of CLUSTAL Omega for sequence alignments and structure. After finishing the alignment, the generated file was viewed using MView, and variations in the sequence or amino acid changes were noted. For the prediction of secondary structures of the SARS-CoV2 spike protein, CFSSP (Chou and Fasman secondary structure prediction) server was used in this experiment. To check for the presence of similarities or differences, all these spike protein sequences were first aligned in CLUSTAL Omega. We found that at position 614, all the isolates from Bangladesh have a common mutation, D614G whereas, in the reference genome (Wuhan), it is ‘D’ instead of ‘G’. So, in this position, all the isolates of amino acid D (aspartic acid) changed into G (Glycine). Also, at position 681, the isolates BCSIR-NILMRC_393, BCSIR-NILMRC_396, BCSIR-NILMRC_422, and BCSIR-NILMRC_424 show the mutation P681R (Fig. 1).

Figure 1 shows the secondary structures in and around the site of the mutations, highlighting the loss of turn in positions 614 and 681. These conformational changes may affect the function of the receptor-binding subunit S1 of the S protein, particularly in the recognition of the host-cell receptor ACE2. Cryo-EM Delta spikes and S-ACE2 complex structures show that RBD destabilizations cause a population shift toward a more RBD-up and S1-destabilized fusion-prone state, which is advantageous for ACE2 engagement and S1 shedding^39^.

**Figure 2:**
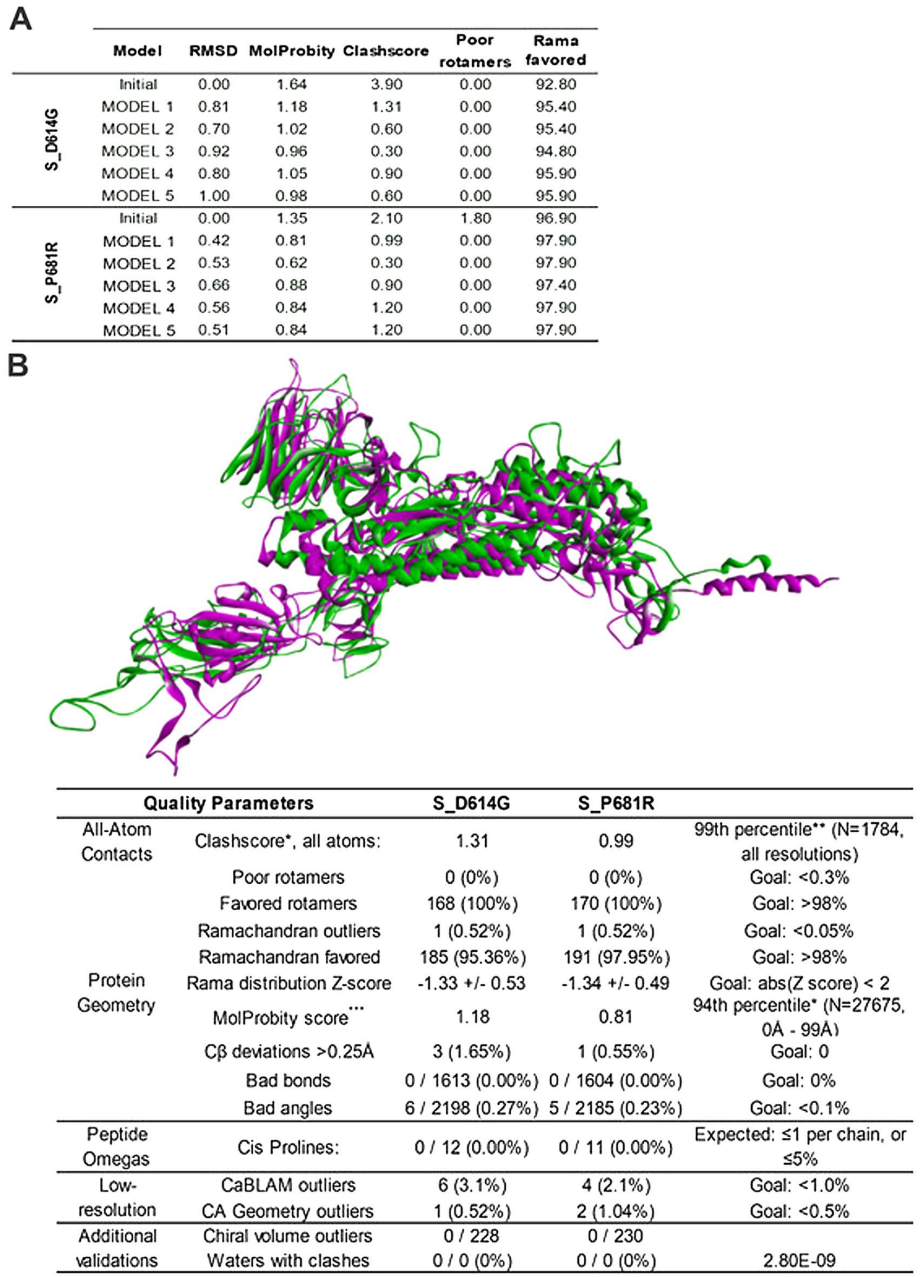
(**A**) The best models generated by the tertiary structure refinement step of the mutant spike proteins. (**B**) 3D conformation, stereochemical, and structural parameters of S_D614G and S_P681R proteins after molecular modeling and refinement.

The amino acid sequences of our isolates were submitted to the Swiss-Model/Raptor X server to construct the tertiary structure of the S proteins with mutations D614G (S_D614G) and P681R (S_P681R). The best model in each case was refined using short molecular dynamics relaxations after repeated side chain repacking perturbations using GalaxyRefine2. Of the 10 models provided to S_D614G and S_P681R, the one with the best stereochemical and structural parameters was selected, considering the analyses of bond length and angle geometry, Ramachandran, rotamer, C_β_ deviation analysis, cis-peptide, CaBLAM analysis and after run clashscore to find bad clashes and clashscore, a saber: RMSD (0.814 and 0.42), MolProbity (1.18 and 0.81), Clash score (1.31 and 0.99), poor rotamers (0 and 0), Ramachandran favored (95.36% and 97.95%), C_β_ deviation (1.65% and 0.55%) and CA geometry outliers (0.52% and 1.04%) (Fig. 2), respectively. The MolProbily score represents the most important protein quality statistic because it combines the Clashscore, Rotamer, and Ramachandran scores into a single normalized score. The RMSD indicates the average deviation between the atoms of the refined and unrefined structures, and this value should be as low as possible.

**Figure 3:**
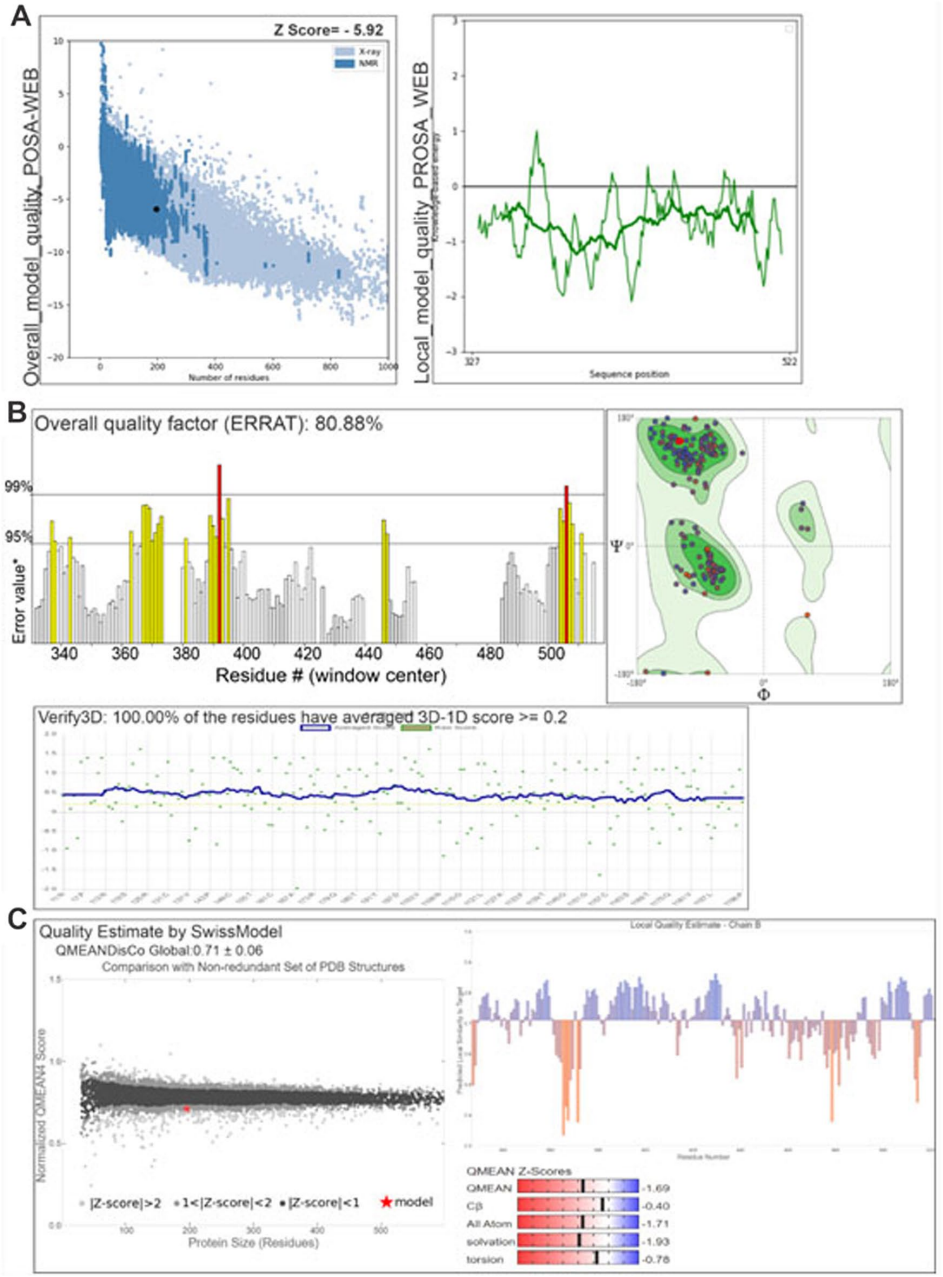
Validation of the final S_D614G protein model. (**A**) ProSA-web showing the Z-score. (**B**) Quality factor and quality score by ERRAT and Verify3D, respectively. (**C**) Information about the global and residual qualities of the protein generated by the Swiss Model’s Structure Assessment tool.

**Figure 4:**
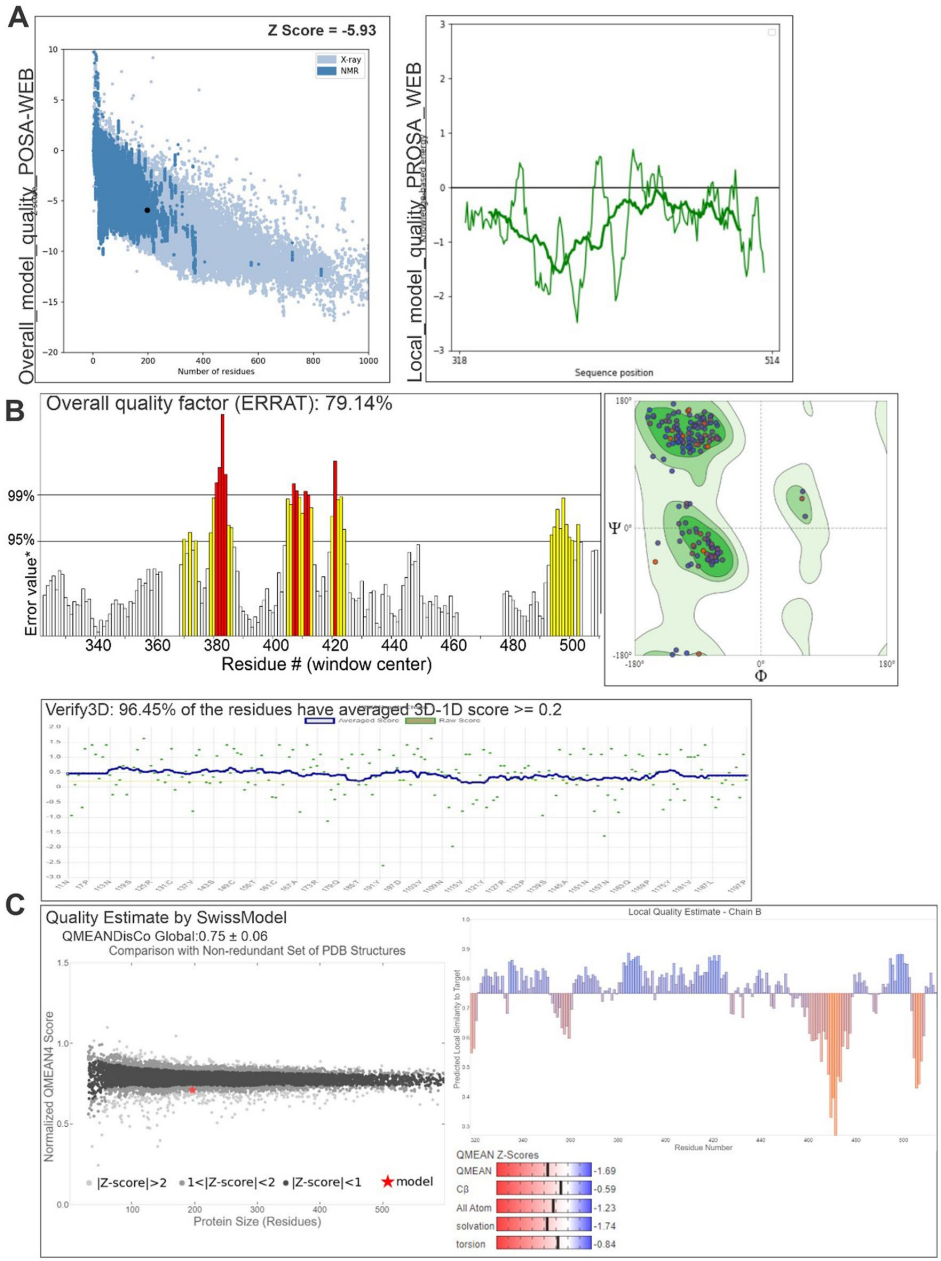
Validation of the final S_P681R protein model. (**A**) ProSA-web demonstrating the Z-score; (**B**) quality factor and quality score by ERRAT and Verify3D, respectively; (**C**) information about the global and residual qualities of the protein generated by the Swiss Model’s Structure Assessment tool.

To verify the structural and stereochemical qualities of the refined models of protein S_D614G (S_P681R), all-atom structure validation analyses were performed using MolProbity, Ramachandran plot, Z-score, ERRAT, Verify3D, and PROCHECK tools (Figs. 3 and 4). According to Ramachandran’s plot, 95.36 percent (97.95 percent) of the residues were in the most favorable ranges, while only 4.12 percent (1.53 percent) were in the allowable ranges. Disallowed ranges make up 0.52 percent (0.52%). The Z-score of the model was estimated to be − 5.92 (− 5.93), which is within the range of scores normally found for native proteins of similar size. According to ERRAT, the overall quality factor of the protein model compared with highly refined structures was 80.88% (79.14%), and in Verify3D, 100.00% (96.45%) of the amino acid residues have an average 3D–1D score >  = 0.2, showing high compatibility with the databases used for comparison. Validation was completed, with no critical errors found in the tertiary structure model.

**Figure 5:**
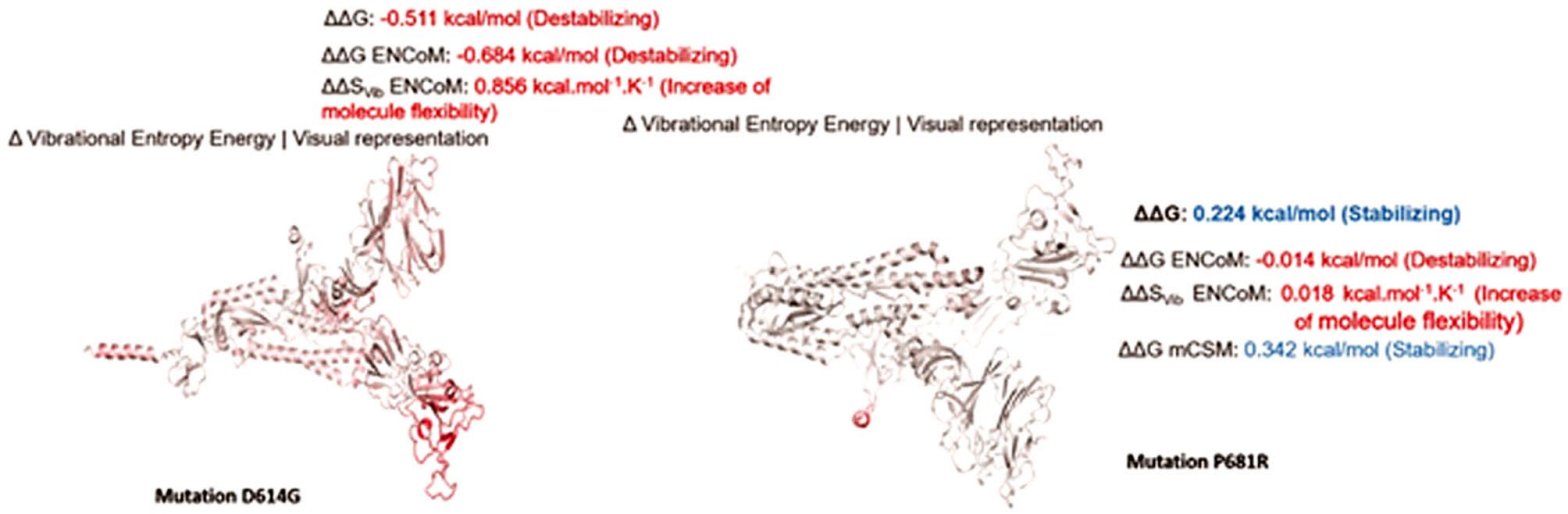
Vibrational Entropy Energy between Wild-Type and Mutant for mutation D614G and P681R in BCSIR-NILMRC-422. Amino acids are colored according to the vibrational entropy change upon mutation. BLUE represents a rigidification of the structure and RED a gain in flexibility.

A point mutation can significantly affect the structure, stability, and flexibility of some proteins by disrupting conformational constraints through overpacking or physicochemical effects. These changes can lead to perturbations in protein function, affect the difference in free energy between the folded and unfolded states of the protein, and cause changes in the interaction energy between residues^40^. To determine if changes in secondary structure are also reflected in the dynamics of the protein in its tertiary structure, we performed normal mode analyses and studied S protein stability and flexibility. The change in vibrational entropy energy (ΔΔSVib ENCoM) in case of mutation D614G between the wild-type Wuhan isolate and the BCSIR-NILMRC- 422 isolate was 0.856 kcal/mol K. It was 0.018 kcal/mol K in case of mutation P681R (Fig. 5). The ΔΔG was − 0.511(0.224) kcal/mol and the ΔΔG ENCoM was − 0.684 (− 0.014) kcal/mol for mutation D614G (P681R). These findings confirmed a stabilizing mutation in these types of spikes.

**Figure 6:**
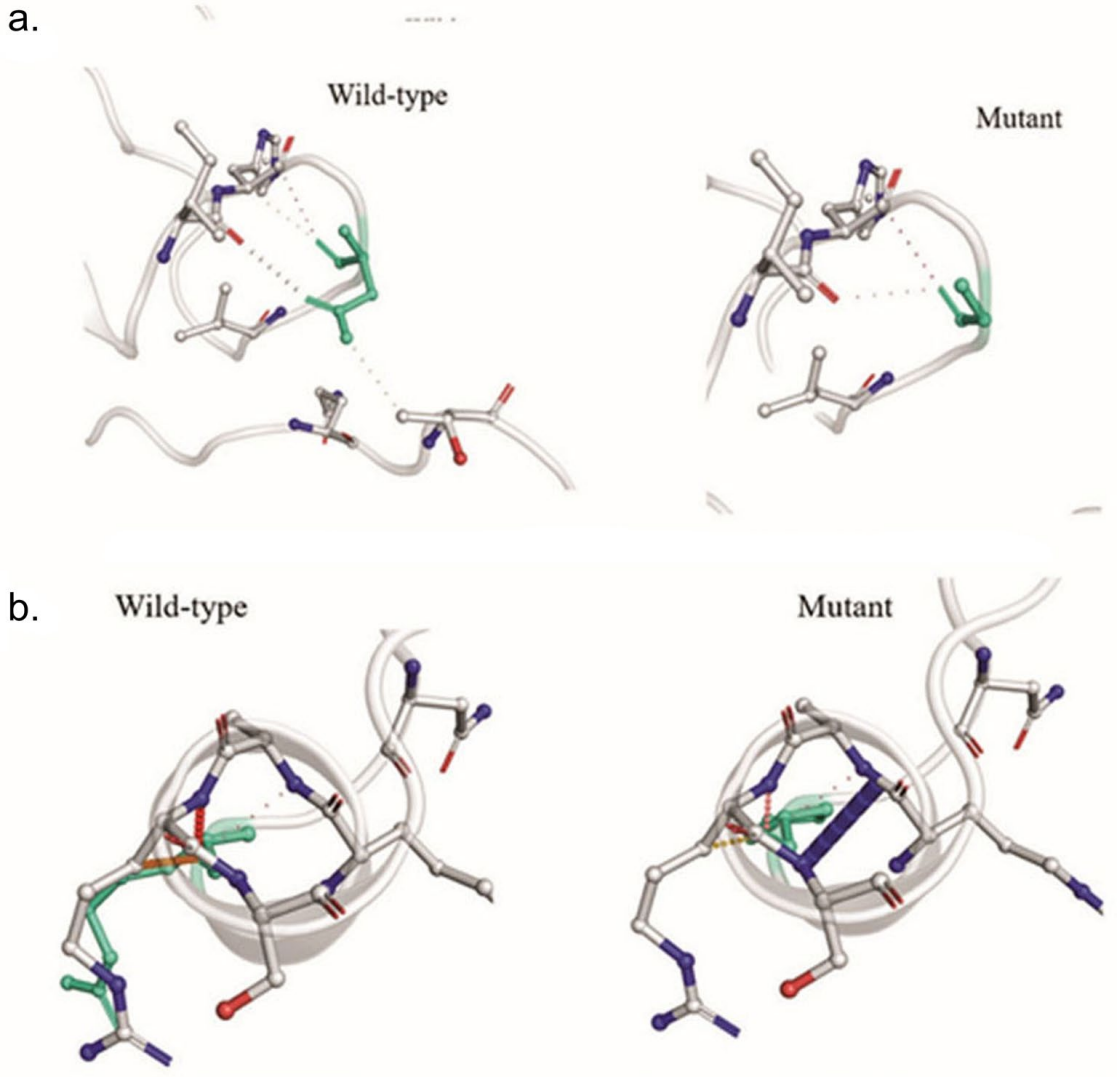
(**a**) Interatomic interaction of D614G in isolate BCSIR-NILMRC-422. Wild-type and mutant residues are colored in light-green and are also represented as sticks alongside the surrounding residues which are involved in any type of interaction. (**b**) Interatomic interaction of mutation P681R in isolates BCSIR-NILMRC-424. Wild-type and mutant residues are colored in light-green and are also represented as sticks alongside the surrounding residues which are involved in any type of interaction.

**Figure 7:**
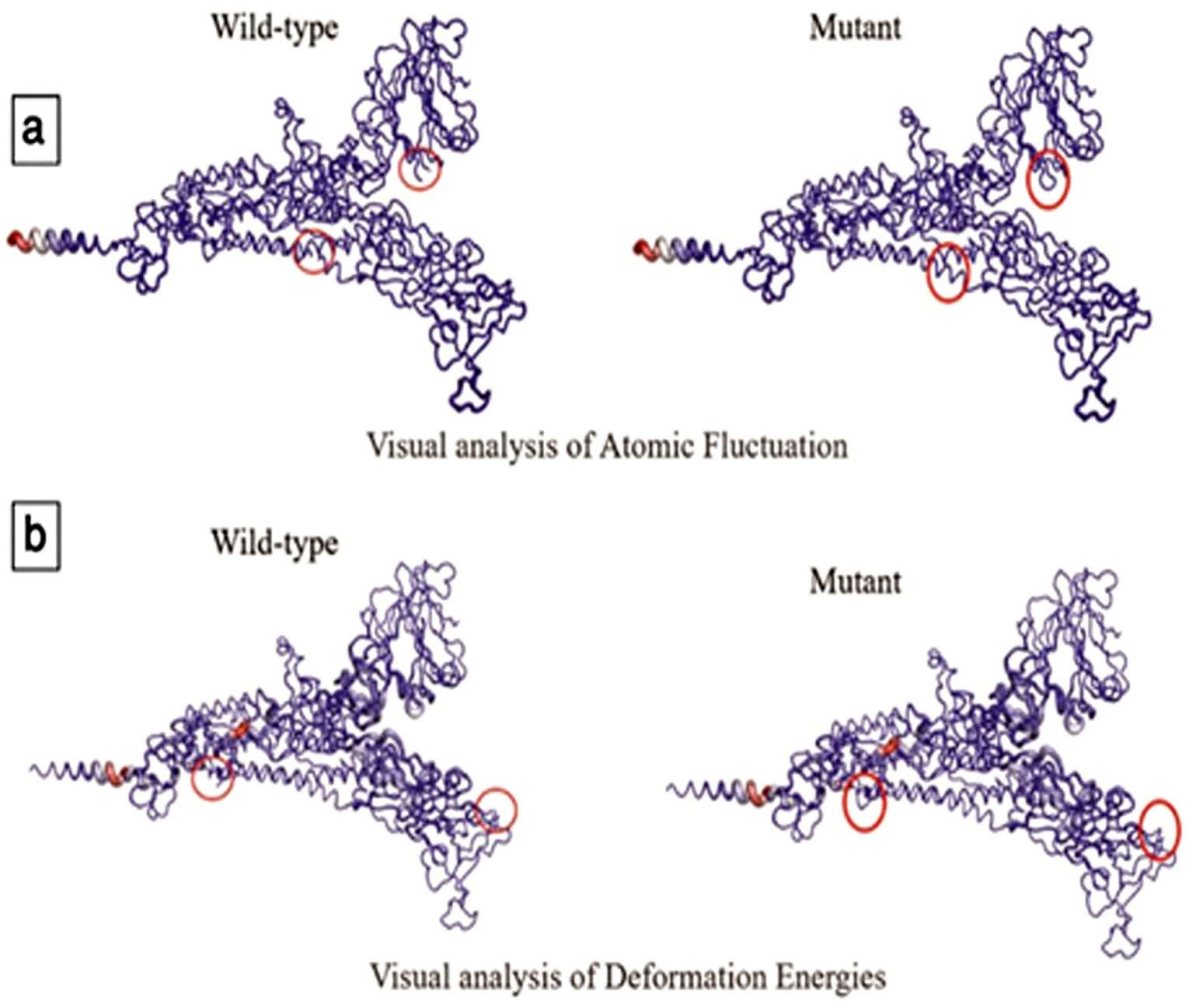
Visual analysis of fluctuations and deformation energies of mutation D614G in BCSIR-NILMRC-422. The magnitude of (**a**) atomic fluctuation and (**b**) deformation has been shown using thin to thick lines colored blue (low), white (moderate), and red (high).

**Figure 8:**
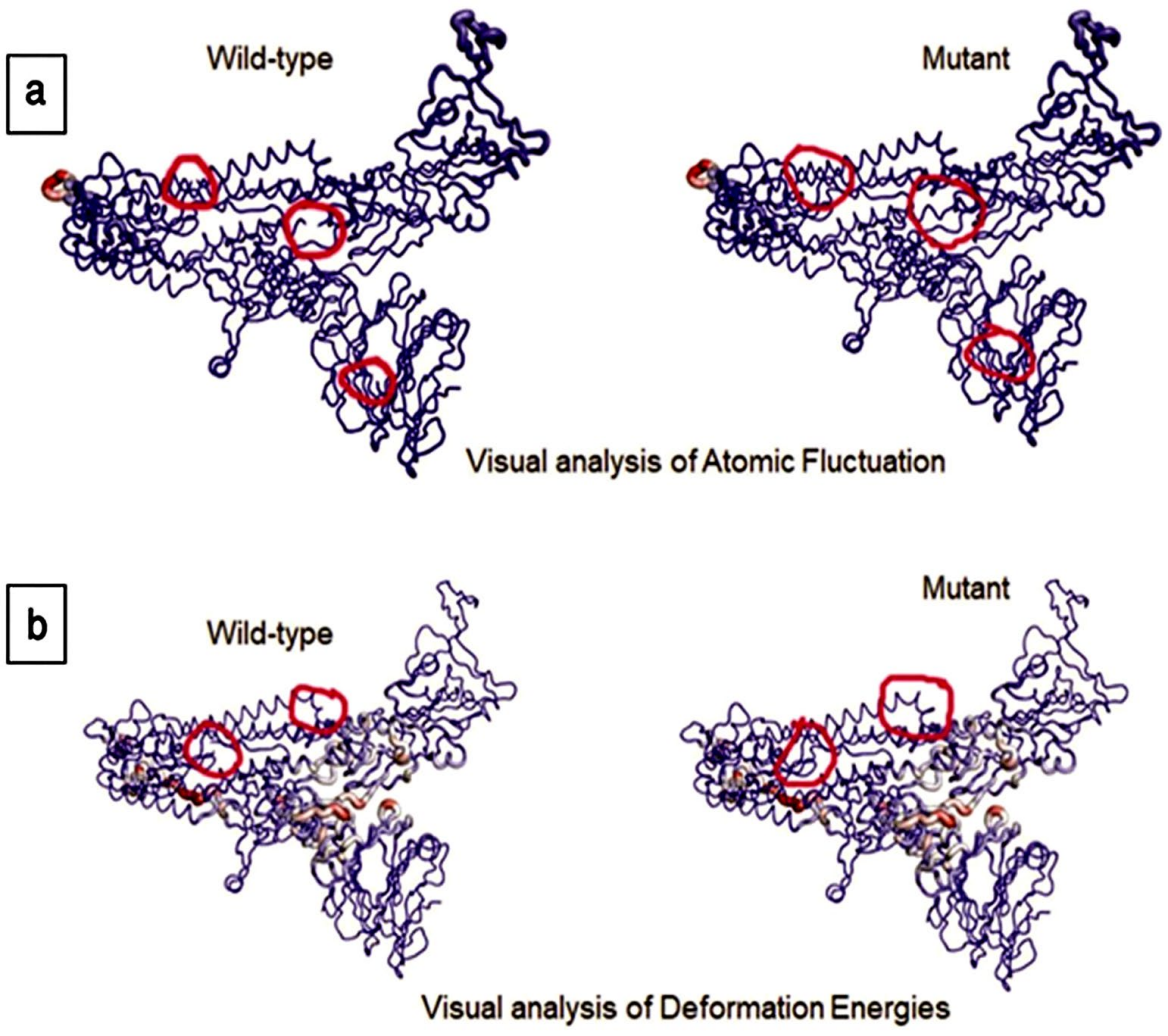
Visual analysis of fluctuations and deformation energies of mutation P681R in BCSIR-NILMRC-424. The magnitude of (**a**) atomic fluctuation and (**b**) deformation has been shown using thin to thick lines colored blue (low), white (moderate), and red (high).

The interatomic interactions of mutations S_D614G and S_P681R have been shown in Fig. 6. We observed significant changes in the positions of these isolates (Figs. 7 and 8). Atomic fluctuations such as these indicate the degree of atomic motion deformation, while energy measurements detect protein flexibility.

Having identified the predominant strains in Bangladesh in 2021 and designed 3D models of the spike proteins of these viruses, we address here the description of the binding modes of their respective RBD with ACE2 by docking molecular and QM/MM simulations. Binding of the S_D614G and S_P681R proteins to ACE2 performed by the Cluspro server resulted in a set of possible models ordered by cluster size. In HADDOCK refinement, a solvent shell was built around the best complexes and, subsequently, a series of short MD simulations were performed according to the parameters below, all atoms except the side-chain atoms at the interface are restrained to their original position. Next, 1250 Molecular Dynamics steps were performed at 300 K with position restraints for heavy atoms which are not part of the PPI (residues not involved in intermolecular contacts within 5 Å). Finally, the systems were cooled down (1000 MD steps at 300, 200 and 100 K) with position restraints on the backbone atoms of the protein complex, excluding the interface atoms.

**Figure 9:**
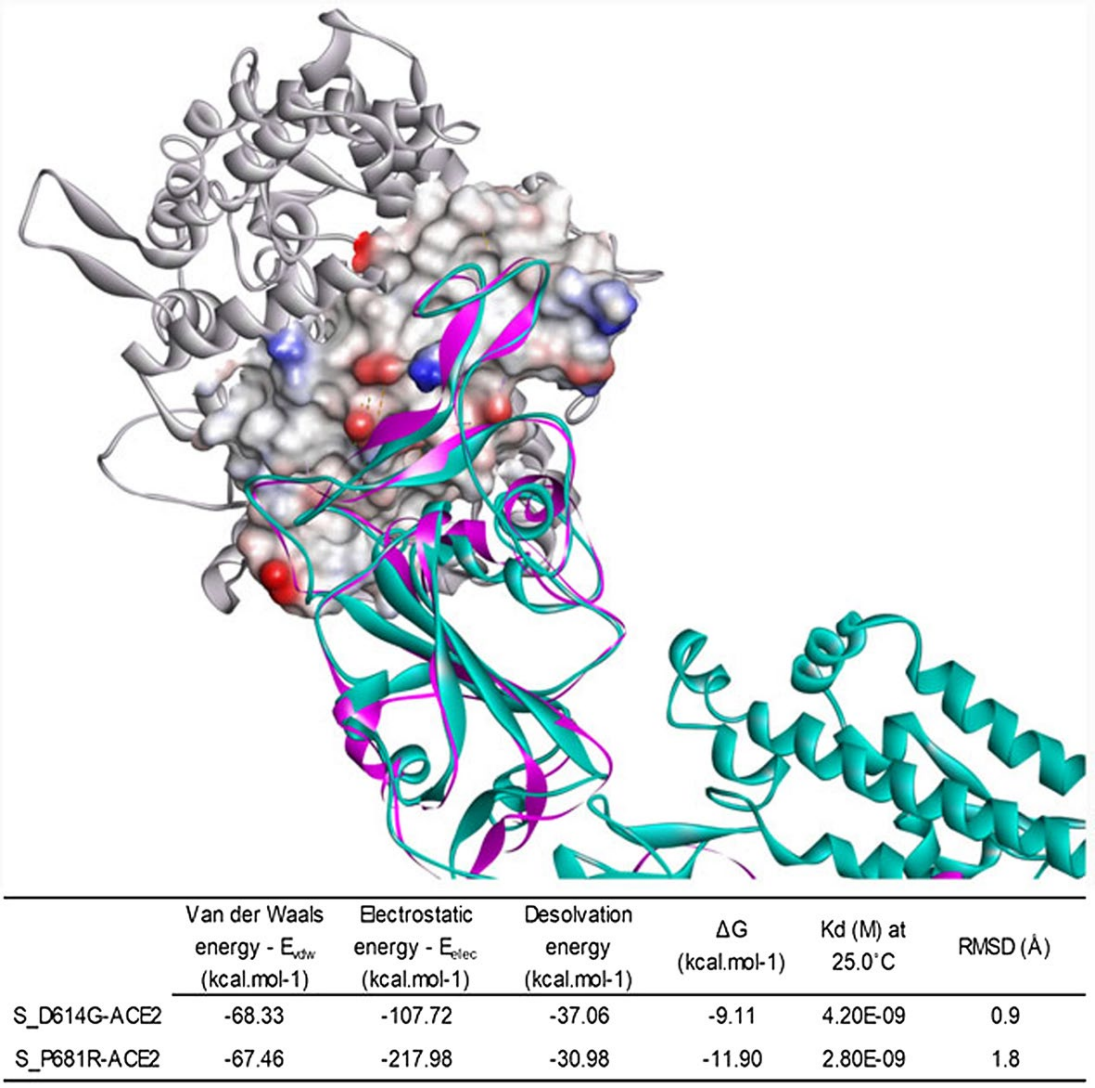
The best 3D docking model and energetic aspects of the S_D614G-ACE2 and S_P681R-ACE2 complexes (superimposed) according to the QM/MM refinement protocol.

The best models were analyzed using the PRODIGY tool (Fig. 9). The predictive model developed in PRODIGY was trained using a subset of bound structures from the structure-based protein–protein binding affinity benchmark consisting of 144 nonredundant protein–protein complexes with known 3D structures (both unbound and bound components) and associated experimental binding affinity values (ΔG)^41^. For S_D614G-ACE2, the QM/MM model had an RMSD of 0.9 Å compared to the Cluspro model, and the ∆G_QM/MM_ (− 9.11 kcal/mol) < ∆G_Cluspro_ (− 8.55 kcal/mol). Similarly, the S_P681R-ACE2 structure showed an RMSD of 1.8 Å and ∆G_QM/MM_ (− 11.90 kcal/mol) < ∆G_Cluspro_ (− 9.24 kcal/mol). The significance of the hybrid geometry optimization protocol (combining classical and quantum theory) is highlighted by these values as it shows the method to be far more precise and dependable^26^. After establishing an interfacial distance of 5.5 Å (10.1002/pro.580), the complex S_D614G-ACE2 (S_P681R-ACE2) has 1 (8), 10 (7), 24 (31), 5 (3), 18 (13), 13 (14) atomic contacts of the charged-charged, charged-polar, charged-apolar, polar-polar, polar-apolar, and apolar-apolar types.

**Figure 10:**
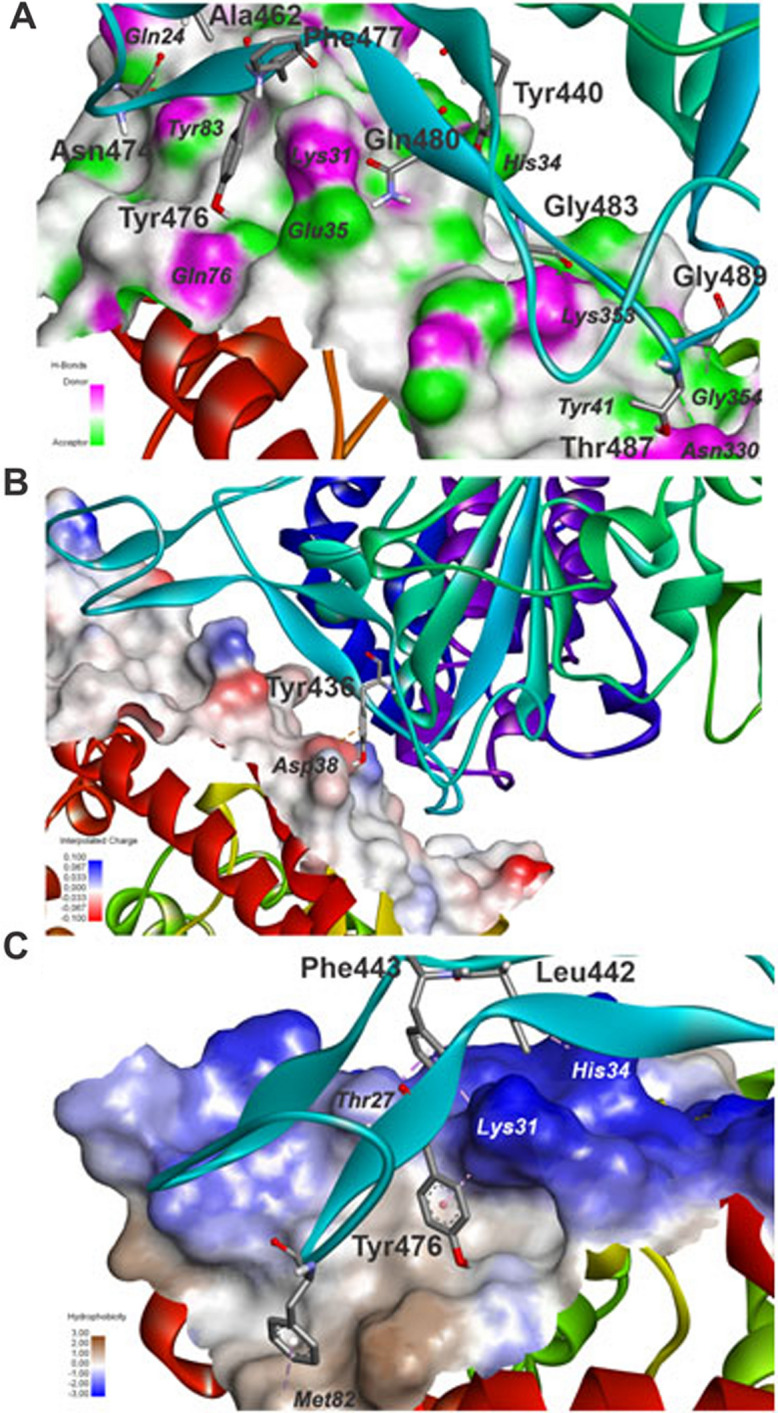
Intermolecular interactions between S_D614G and ACE2 evidenced by receptor surfaces characterized by (**A**) H-bond donor–acceptor, (**B**) interpolated charge, and (**C**) hydrophobicity.

**Figure 11:**
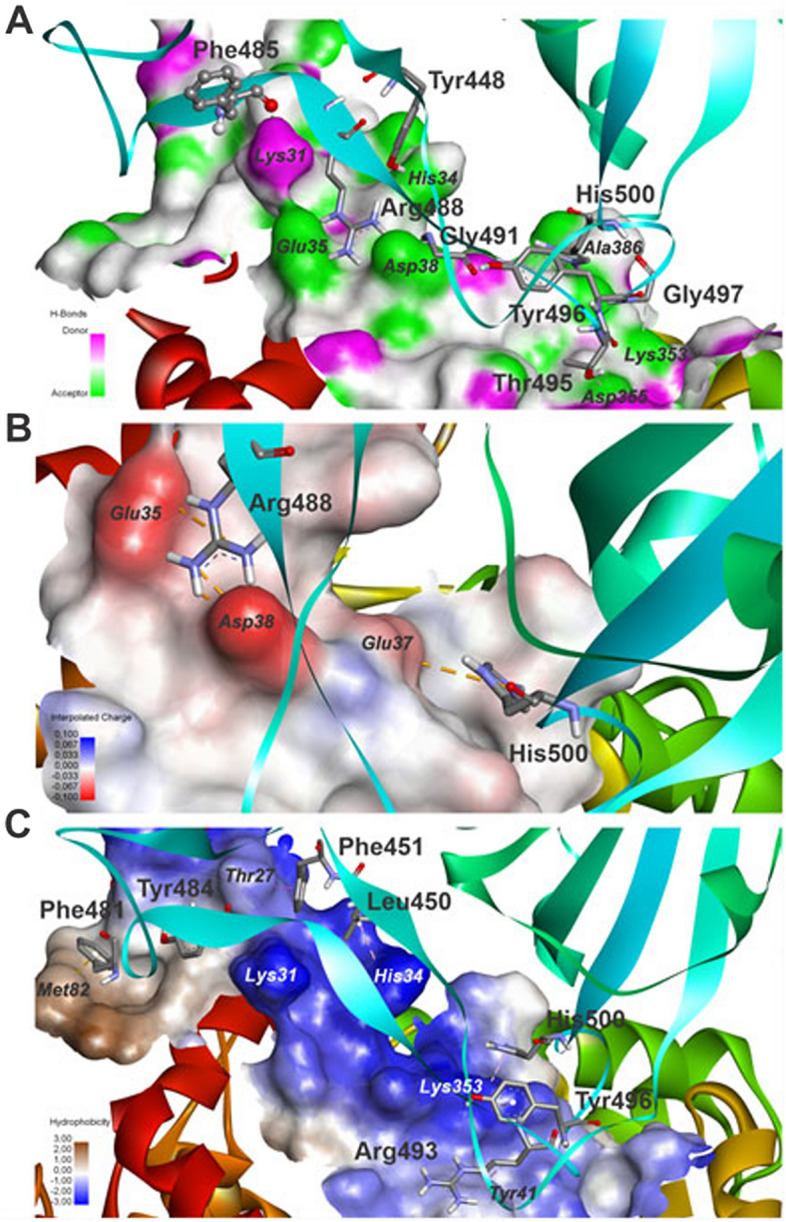
Intermolecular interactions between S_P681R and ACE2 evidenced by receptor surfaces characterized by (**A**) H-bond donor–acceptor, (**B**) interpolated charge, and (**C**) hydrophobicity.

In details, Discovery Studio, LigPlot+ and PoseView were used to extract the graphical image of the S protein-receptor interaction profile of the QM/MM complexes (Figs. 10 and 11). Of the total 18 (19) intermolecular interactions, the S_D614G-ACE2 (S_P681R-ACE2) interface presents 01 (03) salt bridge between TYR436-ASP38_ACE2_ (ARG488-ASP38_ACE2_, ARG488-GLU35_ACE2_ and HIS500-GLU37_ACE2_), 12 (09) hydrogen bonds between ALA462-GLN24_ACE2_, GLN480-LYS31_ACE2_, ASN474-TYR83_ACE2_, THR487-ASN330_ACE2_, GLY483-LYS353_ACE2_, TYR440-HIS34_ACE2_, ASN474-GLN24_ACE2_, TYR476-GLN76_ACE2_, GLN480-GLU35_ACE2_, THR487-TYR41_ACE2_, GLY489-LYS353_ACE2_ and PHE477-LYS31_ACE2_ (PHE485-LYS31_ACE2_, TYR496-LYS353_ACE2_, TYR448-HIS34_ACE2_, ARG488-GLU35_ACE2_, GLY491-ASP38_ACE2_, GLY497-LYS353_ACE2_, HIS500-ALA386_ACE2_, THR495-ASP355_ACE2_ and GLY497-GLY354_ACE2_), and 05 (07) contacts with apolar property between PHE443-THR27_ACE2_, LEU442-HIS34_ACE2_, PHE443-LYS31_ACE2_, PHE473-MET82_ACE2_ and TYR476-LYS31_ACE2_ (PHE451-THR27_ACE2_, LEU450-HIS34_ACE2_, PHE481-MET82_ACE2_, TYR484-LYS31_ACE2_, ARG493-TYR41_ACE2_, TYR496-LYS353_ACE2_ and HIS500-LYS353_ACE2_).

## Discussion

Sequences that are not part of any cluster of concern will be designated as *International AB Diversity*^30,31^. Because of the sequence of a novel coronavirus from Wuhan, the original version of this tool could only be created weeks after the virus was first sequenced. The data generated by the Shanghai Public Health Clinical Center & School of Public Health, the National Institute for Viral Disease Control and Prevention, the Institute of Pathogen Biology, and the Wuhan Institute of Virology and shared via GISAID, was released before publication.

The B.1.351 (501Y.V2) variant, which is our cluster of concern, emerged in late 2020 in the Eastern Cape Province, South Africa, and subsequently spread throughout more than 40 countries worldwide, including Bangladesh. During the first wave of the pandemic, epidemiological and modeling studies showed that the B.1.351 variant was more transmissible compared to other lineages circulating. At present, there is ambiguity concerning the ability of B.1.351 to impact COVID-19 severity. Phylogenetic analysis showed that all the cases of SARS-CoV-2 belong to the GR Clade.

Before analyzing the single nucleotide polymorphism of our genomes isolated from Bangladeshi patients and discussing the implications of these changes for SARS-CoV-2 virus pathogenicity, it is important to understand the mechanism of viral infection, which begins with the binding of viral particles to cellular receptors on the host surface. As with other coronaviruses, the spike (S) glycoprotein of SARS-CoV-2 is a membrane fusion machinery that mediates receptor recognition and virus entry into cells and is the primary target of the humoral immune response during infection^42^. The ectodomain of the S protein consists of a receptor-binding subunit S1 and a membrane-fusing subunit S2. Two major domains have been identified in coronavirus S1, an N-terminal domain (NTD) and a C-terminal domain (CTD), which is also referred to as the receptor-binding domain (RBD)^43,44^. The latter is the one that binds to the host cell receptor, human angiotensin-converting enzyme 2 (ACE2)^4^.

In the S1 domain, the most significant mutation was D614G. This mutation, located near the receptor-binding domain at a downstream position, emerged in the spring of 2020 and quickly became predominant^45^. The D614G-bearing variant has quickly swept out the original strain because the D614G mutation increases viral infectivity, fitness, and inter-individual transmissibility^37^.

Another mutation, P681R, is harbored further downstream in the S protein in the same receptor-binding domain. The P681R mutation in the furin cleavage site is known to enhance the basicity of polybasic stretching and the likely facilitation of additional contacts with furin for S1–S2 cleavage^46^. This could help with an increased rate of membrane fusion, internalization and thus better transmissibility. In fact, Mlcochova et al.^47^ showed that B.1.617.1 and B.1.617.2 spike proteins mediated higher fusion activity and syncytium formation than WT, probably mediated by P681R. The P681R mutation apparently caused a small increase in proteolytic processing that could affect infectivity^48^. Reversal of the P681R mutation to wild-type P681 significantly reduces replication of the delta variant to a level below that of the alpha variant^49^.

To predict the binding affinity and to understand the intermolecular interactions das proteínas virais mutantes com o receptor ACE2, protein–protein docking was implemented. The best models were selected, refined using our robust HADDOCK and QM/MM protocol [^11,12^, 10.1016/j.carbpol.2016.06.044, 10.1016/j.meegid.2021.104826].

The QM/MM method has several advantages over other computational methods, in particular because of its ability to accurately describe the electronic structure of a system. In drug/vaccine design, QM/MM calculations are used to investigate the mechanisms and binding modes of drug/prototype candidates, their binding affinity, and interactions with the target protein^26,27^. Here, significant features were found among the total 18 (19) intermolecular interactions of the QM /MM structure of S_D614G-ACE2 (S_P681R-ACE2). We believe that the increase in affinity of the spike protein of SARS-CoV-2 virus with the P681R mutation for the human ACE2 receptor is due to strong electrostatic forces between the ARG488_ACE2_ residue with Glu35 and Asp38 and between HIS500_ACE2_ and GLU37, confirming the electrostatic energy values of S_D614G-ACE2 (− 107.72 kcal/mol) and S_P681R-ACE2 (− 217.98 kcal/mol). Interestingly, HIS500RBD in SARS-2 (alpha variant) is involved in repulsion with Y41 through the oxygen atoms of its side-chain hydroxyl groups [10.1021/acs.jcim.1c01544]. To date, there are no reports in the literature on the importance of ARG488ACE2 for the infectivity of the S:P681R SARS-CoV-2 variant.

The comparison of the interaction profile of the complexes reveals the shared importance of residues LYS31_ACE2_, HIS34_ACE2_, TYR41_ACE2_, GLU35_ACE2_, ASP38_ACE2_, and LYS353_ACE2_ for binding the two spike proteins. These results agree well with previous experimental and computational data [^18^, 10.1038/s41598-020-73820-8]. Recently, Net et al., used quantum biochemical techniques in the framework of density functional theory (DFT) and the molecular fragmentation with conjugated caps (MFCC) approach to analyze the interactions between ACE2 and the spike protein of SARS-CoV-2 (alpha variant) and map the hot-spot residues that form the recognition surface. Residues Q24_ACE2_, T27_ACE2_, F28_ACE2_, D30_ACE2_, K31_ACE2_, H34_ACE2_, E37_ACE2_, D38_ACE2_, Y41_ACE2_, Q42_ACE2_, and L45_ACE2_ account for about 69% of the total interaction energies between the spike protein and ACE2, making 09, 08, 08, 07, 14, 11, 06, 11, 12, 06, 05 interaction pars, respectively [10.1021/acs.jcim.1c01544]^50^. An interesting study using fragment molecular orbitals was performed to characterize protein–protein interactions (PPI) at RBD-ACE2 interface. Herein, four residues (E37_ACE2_, K353_ACE2_, G354_ACE2_, and D355_ACE2_) of ACE2 were identified as strongly interacting with the spike proteins of SARS-CoV-2 [10.1038/s41598-020-73820-8].

Unfortunately, few papers evaluating PPI between mutant spike protein and ACE2 have examined the viral genomic variants prevalent in the Bangladeshi population. Through mutagenesis studies on spike proteins, Yi et al., reported that mutations in residues N501, Q498, E484, T470, K452, and R439 dramatically reduced binding affinity to ACE2 [10.1038/s41423-020-0458-z]. Zou et al., performed molecular dynamics simulation along with alanine scanning analysis and found that the relative free energy of binding of the ACE2/SARS complex changed significantly when a mutation was present in spike residues R426, L443, Y484, and T487 [10.1021/acs.jcim.0c00679]. Rawat et al., examined the conserved residues in the spike protein by in silico analysis and showed that the conserved glycine and tyrosine residues in SARS-2 (G502 and Tyr449) are important for both stabilization of the spike protein and its interaction with ACE2 [10.1002/prot.26024].

Our study offers significant insights into the genetic epidemiology of SARS-CoV-2 in Bangladesh, focusing on the identification of dominant variants and associated mutations, with implications for surveillance, diagnostic strategies, and therapeutic interventions. Continuous genomic surveillance, underscored by our identification of the D614G and P681R mutations in the spike protein, is crucial for monitoring viral evolution and managing the severity of infections^51^. Future diagnostic strategies could leverage these specific mutations to develop novel tools for accurate tracking and early intervention. In terms of therapeutic interventions, these mutations, which potentially enhance the binding affinity between the spike protein and the ACE2 receptor, could guide the design of treatments that target these mutations and inform vaccine development to ensure effectiveness against prevalent variants. Future research should explore the impact of these mutations on the virus's transmissibility, disease severity, and their potential effect on the effectiveness of current vaccines and treatments, which will be pivotal in developing strategies to combat COVID-19.

## Conclusion

The presence of the highly conserved mutation P681R in four out of seventeen Bangladeshi samples of SARS-CoV-2 indicates an enhancement in viral fusion efficiency and an accelerated action speed. In this study, we utilized the Dynamut web server, an integrated computational method, for stability prediction. Dynamut enables the simulation of protein dynamics and the prediction of protein stability, providing a consensus approach for accurate and reliable prediction of both stabilizing and destabilizing mutations at positions 614 and 681. Although the exact impact of the P681R mutation and its relationship with COVID-19 severity and unusual symptoms caused by SARS-CoV-2 infection are yet to be fully understood, detailed investigations on this mutation are planned. Further research will contribute to improved therapeutic targeting and the development of in silico vaccine designs. Due to the potential association of the P681R mutation in the spike protein with viral infection severity or unusual outcomes, it is crucial to conduct intensive analysis of SARS-CoV-2 variants harboring this mutation.

This study presents the first documented cases of the P681R mutation in Bangladesh, specifically in the isolates BCSIR-NILMRC-393, BCSIR-NILMRC-393, BCSIR-NILMRC-422, and BCSIR-NILMRC-424. Further sequencing efforts, accompanied by comprehensive annotation, will provide deeper insights into the spike protein mutation of SARS-CoV-2 in humans. These variations may contribute to the diversification of the virus and the subsequent emergence of variants, strains, serotypes, and antibody escape mutants^52^. Moreover, these mutations could aid the virus in adapting better to the host environment and expanding its tissue tropism. The increased affinity between the spike protein of SARS-CoV-2 carrying the P681R mutation and the human ACE2 receptor is attributed to strong electrostatic forces involving the ARG488 residue with Glu35 and Asp38, as well as interactions between His500 and GLU37.

### Supplementary Information

Supplementary Figure 1.

Supplementary Figure 2.

Supplementary Legends.

## Data Availability

The sequences of SARS-CoV-2 genome from Bangladesh were submitted in the GISAID database accession no. EPI_ISL_603221, EPI_ISL_603222, EPI_ISL_603223, EPI_ISL_603224, EPI_ISL_603225, EPI_ISL_603238, EPI_ISL_603239, EPI_ISL_603240, EPI_ISL_603241, EPI_ISL_603242, EPI_ISL_603243, EPI_ISL_603244, EPI_ISL_603245, EPI_ISL_603246, EPI_ISL_603247, EPI_ISL_603249, EPI_ISL_603250. SRA number: BCSIR-NILMRC_422 SRA- SRP336906 (PRJNA762998), BCSIR-NILMRC_424 SRA- SRP336782 (PRJNA762670).

## Abbreviations

SARS-CoV-2: Severe acute respiratory syndrome coronavirus 2
NCBI: National Center for Biotechnology Information
IEDB: Immune Epitope Database
GC: Guanine-cytosine
CFSSP: Chou and Fasman secondary structure prediction
NGS: Next Generation Sequencer
MSA: Multiple sequence alignment

## Code availability

The software used : https://coronavirus.jhu.edu/map.html. https://www.who.int/emergencies/diseases/novel-coronavirus-2019. https://corona.gov.bd/?gclid. https://basespace.illumina.com. https://eztaxon-e.ezbiocloud.net/tools/sc2?id=5bbb155a-dd3e-4e9b-9b10-54b4e67926bd. https://swissmodel.expasy.org/interactive. https://cov-lineages.org/lineage.html?lineage=B.1.1.25. https://www.ncbi.nlm.nih.gov/nuccore/NC_045512.2. https://www.ezbiocloud.net/tools/sc2/?id=4024a5b2-40bb-40b5-acd6-c4dd65b38d76. https://outbreak.info/situation-reports?pango=B.1.1.25&muts=S%3AP681R&selected=BGD&loc=USA&loc=USA_US-CA&loc=BGD. https://www.britannica.com/science/amino-acid/Standard-amino-acids. http://www.biogem.org/tool/chou-fasman. https://www.britannica.com/science/amino-acid/Standard-amino-acids. http://biosig.unimelb.edu.au/dynamut/results_prediction.
