# Supplementary figures and images for: Spike protein mutations and structural insights of pangolin lineage B.1.1.25 with implications for viral pathogenicity and ACE2 binding affinity
Source: Sci Rep. 2023 Aug 12;13:13146. doi: 10.1038/s41598-023-40005-y (PMC10423208; doi:10.1038/s41598-023-40005-y)

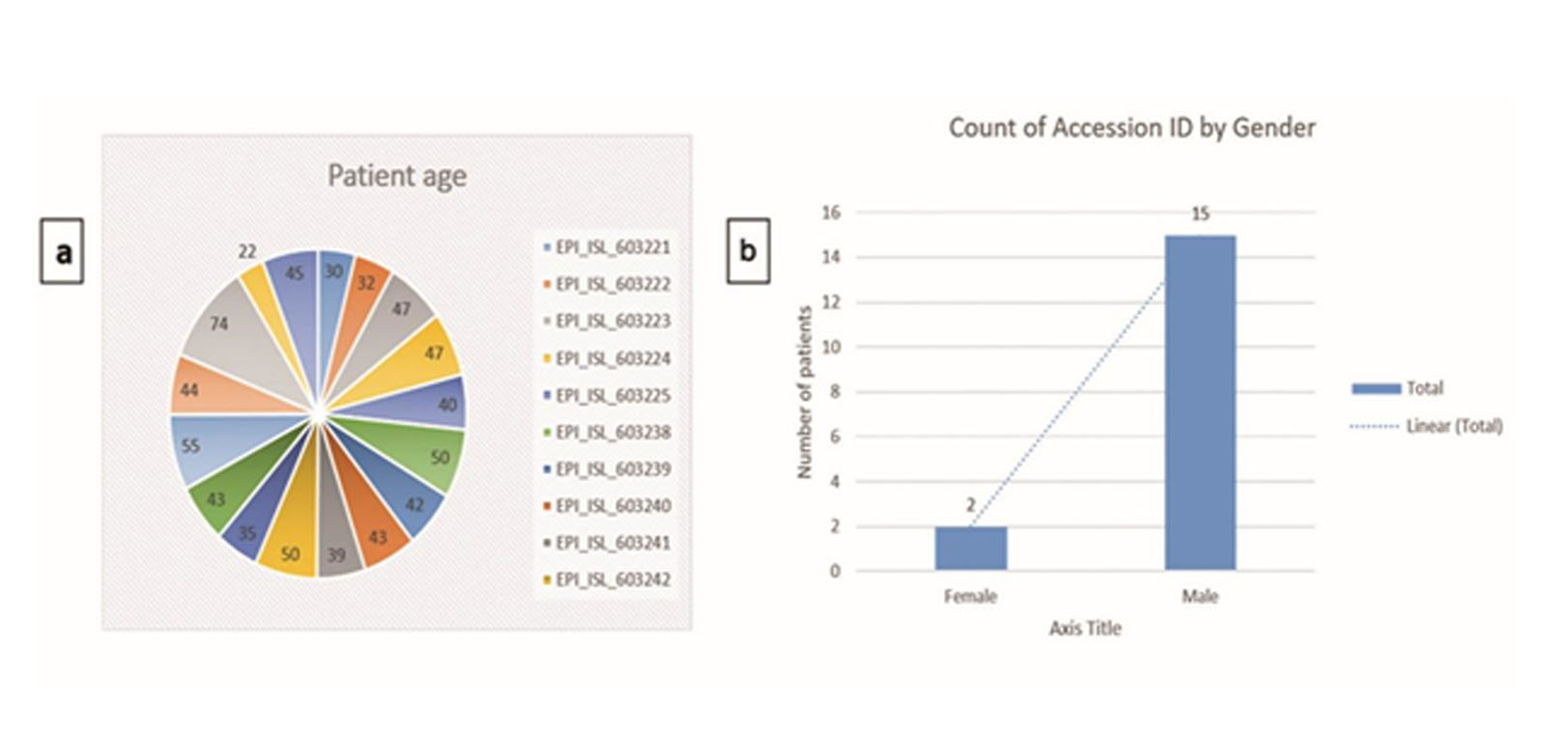

Supplement: Supplementary file 1 — Supplementary Figure 1. [file 41598_2023_40005_MOESM1_ESM.jpg]

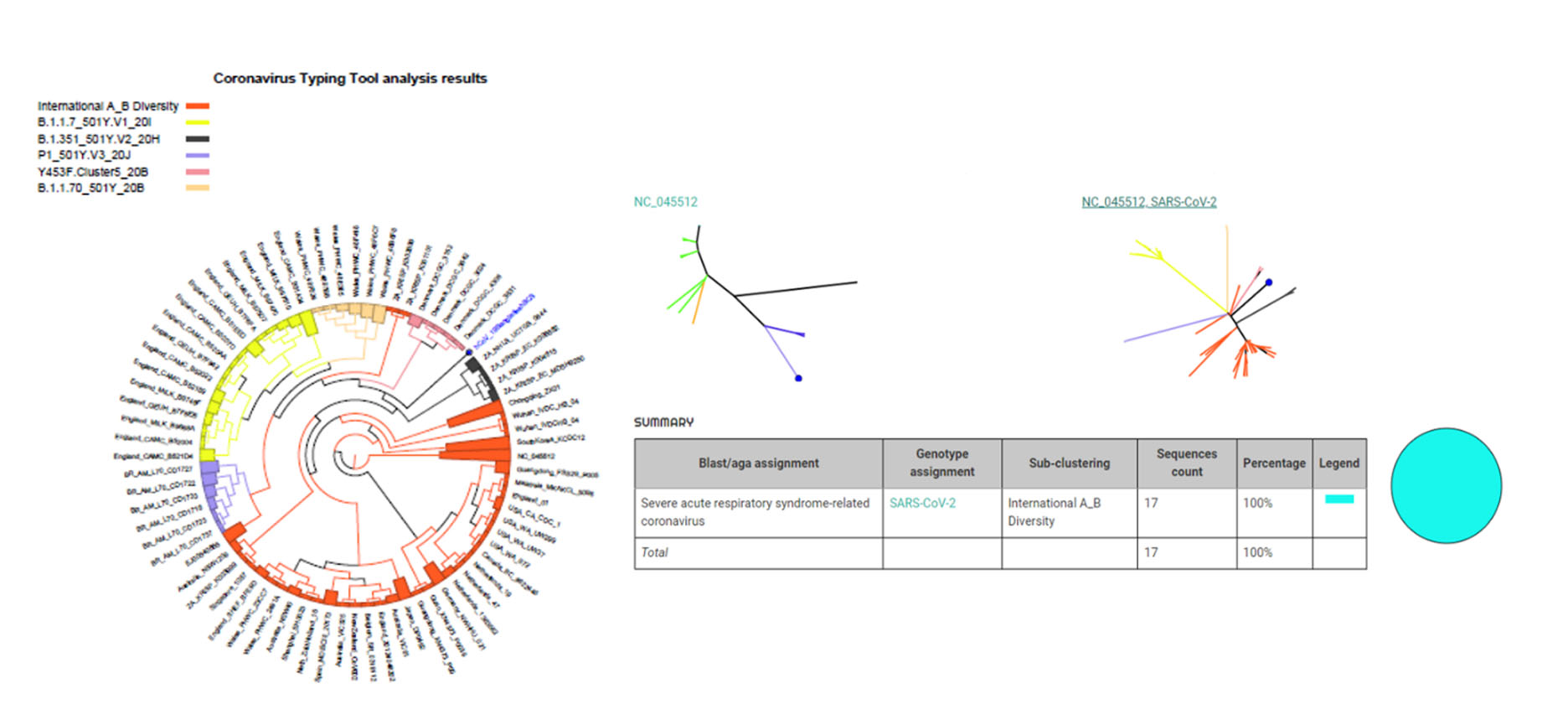

Supplement: Supplementary file 2 — Supplementary Figure 2. [file 41598_2023_40005_MOESM2_ESM.jpg]
