# Supplementary material for: Spike protein mutations and structural insights of pangolin lineage B.1.1.25 with implications for viral pathogenicity and ACE2 binding affinity
Source: Sci Rep. 2023 Aug 12;13:13146. doi: 10.1038/s41598-023-40005-y (PMC10423208; doi:10.1038/s41598-023-40005-y)
Supplement: Supplementary file 3 — Supplementary Legends. [file 41598_2023_40005_MOESM3_ESM.docx]

**Supplementary Figure legends:**

Supplementary Fig. 1: a) The age and b) Sex of the 17 Bangladeshi patients from whom the SARS-CoV2 virus isolates were taken.

Supplementary Fig. 2: Schematic polar diagram displaying the phylogenetic tree of the 17 isolates of SARS-CoV2 sequenced in BCSIR, Bangladesh and comparing with the reference sequence NC_045512. The FASTA file was uploaded to the Genome Detective Coronavirus Typing Tool version 1.17. The toolkit uses BLAST and phylogenetic methods to assign a lineage to the virus sequence according to a method described by Cleemput et al. The genome of interests are of the B.1.351_501Y.V2_20H lineage according to Coronavirus Typing Tool (<https://www.genomedetective.com/app/typingtool>)
